# Genetic influence on brain volume alterations related to self-reported childhood abuse

**DOI:** 10.3389/fnins.2022.1019718

**Published:** 2022-09-20

**Authors:** Tian Tian, Yuanhao Li, Jia Li, Guiling Zhang, Jian Wang, Changhua Wan, Jicheng Fang, Di Wu, Yiran Zhou, Yuanyuan Qin, Hongquan Zhu, Dong Liu, Wenzhen Zhu

**Affiliations:** Department of Radiology, Tongji Hospital, Tongji Medical College, Huazhong University of Science and Technology, Wuhan, China

**Keywords:** childhood abuse, polygenic risk score (PRS), gene expression, neuroimaging, whole exome sequencing (WES)

## Abstract

As an important predictor of adulthood psychopathology, self-reported childhood abuse appears heritable and is associated with brain abnormalities. However, the specific genetic mechanisms behind these brain alterations remain largely unknown. This study recruited young adults who reported different degrees of childhood abuse from the community. In order to fully understand the influence of genes on brain changes related to self-reported childhood abuse, various experiments were conducted in this study. Firstly, volume changes of gray matter and white matter related to childhood abuse were investigated by using advanced magnetic resonance imaging techniques. After sequencing the whole exons, we further investigated the relationship between polygenic risk score, brain volume alterations, and childhood abuse score. Furthermore, transcription-neuroimaging association analysis was used to identify risk genes whose expressions were associated with brain volume alterations. The gray matter volumes of left caudate and superior parietal lobule, and white matter volumes of left cerebellum and right temporal lobe-basal ganglia region were significantly correlated with the childhood abuse score. More importantly, brain volume changes mediated the influence of polygenic risk on self-reported childhood abuse. Additionally, transcription-neuroimaging association analysis reported 63 risk genes whose expression levels were significantly associated with childhood abuse-related brain volume changes. These genes are involved in multiple biological processes, such as nerve development, synaptic transmission, and cell construction. Combining data from multiple perspectives, our work provides evidence of brain abnormalities associated with childhood abuse, and further indicates that polygene genetic risk and risk gene expression may affect the occurrence of childhood abuse by brain regulation, which provides insights into the molecularpathology and neuromechanism of childhood adversity. Paying attention to the physical and mental health of high-risk children may be a fundamental way to prevent childhood abuse and promote lifelong mental health.

## Introduction

Adversity has cumulative effects on cognitive and emotional functions throughout the whole life cycle, but the initial stage of human life is particularly sensitive to adverse conditions. In recent years, childhood maltreatment has aroused widespread concern in the social and scientific fields. In addition to being highly prevalent, it has also been confirmed as a powerful and important predictor of poor health outcomes in adulthood (Copeland et al., 2018; Appelmann et al., 2021), such as borderline personality disorder (Marchetti et al., 2021), posttraumatic stress disorder (Bremner et al., 1997), major depression (Medeiros et al., 2020), anxiety (King, 2021), panic disorder (Quagliato et al., 2021), and alcoholism (Fenton et al., 2013). Much evidence indicates neurobiological mechanisms by which childhood maltreatment increases the vulnerability to psychopathology (McCrory et al., 2012). Genomic brain regulation is considered to be one of the important reasons why childhood abuse increases the lifelong risk (Lutz et al., 2021). Neuroimaging studies of adults have provided evidence of extensive brain abnormalities associated with childhood maltreatment (Lim et al., 2018). However, the specific genetic mechanisms behind brain alterations are still unclear. It is very important for timely and effective intervention in childhood maltreatment and related psychopathology.

Recently, behavior genetics has explained the heritability of self-reported childhood maltreatment through potential gene-environment correlation mechanisms (Dalvie et al., 2020). However, a single nucleotide polymorphism (SNP) or gene can only explain a small part of phenotypic variation. It is insufficient to reflect complex traits of the self-reported childhood maltreatment. According to the polygenic genetic mechanism, the genome-wide association study (GWAS) has brought new hope for early identification of individuals at risk of childhood maltreatment (Dalvie et al., 2020). Polygenic risk score (PRS) is a commonly used method for GWAS follow-up analysis. It is calculated according to the weighted accumulation of thousands of risk mutations carried by individuals, and aims to quantify the cumulative effects of a large number of SNPs or genes (Dudbridge, 2013). With the deepening of GWAS research and application transformation research, the application of the PRS model in risk assessment of common diseases has been verified (Dai et al., 2019; Boccanelli and Botta, 2021; Lu et al., 2021). However, at present, the clinical research of PRS in self-reported childhood maltreatment is still lacking.

Although GWAS has determined the risk loci of self-reported childhood maltreatment, epigenetic mechanisms associated with brain micro-changes remain largely unknown. Evidence indicated that anatomical variations in gene expression closely follow the changes of brain connectional architecture (Fornito et al., 2019). Continuous progress in neuroimaging and genome technologies has promoted the development of transcription-neuroimaging association analysis. Different from genetic variations that GWAS pays attention to in a large sample, the transcription-neuroimaging association study identifies genes whose expression levels are associated with neuroimaging changes in disorders. Based on gene expression data derived from the Allen Human Brain Database (AHBA^1^), several studies have identified risk genes whose expression are associated with altered brain structure and function in neuropsychiatric diseases (Ji et al., 2021; Ma et al., 2021). Bridging the gap between microscopic and macroscopic changes is critical for understanding the pathophysiological mechanisms of self-reported childhood maltreatment. However, no transcription-neuroimaging association study has been performed to identify the expression of genes associated with brain alterations in subjects with a history of childhood maltreatment experience.

The brain itself is a very complex regulatory system. It is difficult to clarify the essence of childhood maltreatment from single and limited information. How to integrate neuroimaging and genetic information, and explore its significance in clinical and public health fields, has become a research hotspot in recent years. Previous studies have seldom focused on polygenic risk and transcription-neuroimaging association analysis synchronously. In this study, 216 young adults who reported different degrees of childhood abuse were recruited from the community. We combined PRS correlation analysis based on whole exome sequencing and transcription-neuroimaging association study to give a more integrative understanding of genetic influence on brain volume alterations related to self-reported childhood abuse. This study aims to: (a) explore brain volume alterations related to self-reported childhood abuse, (b) quantify the relationship between PRS, brain volume alterations, and childhood abuse, and (c) identify childhood abuse-related risk genes whose expressions are associated with brain volume alterations.

## Materials and methods

### Participants

In this study, a total of 216 community samples (aged between 20 and 30 years) with different degrees of childhood abuse were enrolled during the period from 2016 to 2018. All subjects met the criteria provided in Supplementary material, and completed MRI scans, venous blood collection, and questionnaires. The human experiment was approved by the Ethical Committee of Tongji Hospital of Tongji Medical College of Huazhong University of Science and Technology. All subjects signed informed consent forms before participating in this study. The subjects’ consent was obtained according to the Declaration of Helsinki.

### Questionnaires

For the childhood abuse phenotype, we adopted the Chinese version of Childhood Trauma Questionnaire (CTQ)-Short Form to evaluate the emotional abuse, sexual abuse, and physical abuse suffered in childhood (Bernstein et al., 1997; Everaerd et al., 2016). Participants respond to each item using a 5-point scale, and scores range from 1 (never true) to 5 (very often true). Then, according to the counts of the three categories of abuse listed above, an overall childhood abuse count score was constructed.

### Genotyping and polygenic risk score calculation

Detailed genotyping analysis was provided in Supplementary material. Briefly, genomic DNA was extracted from each subject by using DNeasy Blood & Tissue Kit. Exome capture was performed using the SureSelect Human All Exon V6 (Agilent Technologies, Santa Clara, CA, USA). The quantity of libraries was evaluated by Qubit^®^ 2.0 Fluoromete. The quality and size of libraries were estimated by 2100 Bioanalyzer High Sensitivity DNA Assay according to the reagent kit guide. Finally, we retained 44949 SNPs for advanced analysis after excluding SNPs with a call rate < 90%, minor allele frequency < 5%, and Hardy-Weinberg equilibrium *P*-value < 10^–6^.

Due to the quality of the blood sample, 39 subjects did not complete the whole exome sequencing. Then, the PRS of 177 participants was calculated from published GWAS studies on self-reported childhood abuse (Dalvie et al., 2020) by using the allele count model (Dudbridge, 2013). In the calculation of PRS, a single threshold will inevitably lead to false positives or false negatives. On one hand, we calculated a best-fit PRS based on the convincing threshold (*P*_T_ = 0.0354) proposed by Dalvie et al. (2020), which was obtained from the association analysis of big database to predict childhood abuse status and had a good application value. The best-fit PRS represented cumulative effects of SNPs that are most predictive for self-reported childhood abuse. On the other hand, we calculated a total PRS based on all SNPs detected by our whole exome sequencing. In brief, both a best-fit PRS (at *P*_T_ = 0.0354) and a total PRS based on all SNPs were applied in the following analysis, which can make our results more objective and reliable.

### MRI acquisition and data preprocessing procedures

All scans were carried out on a 3.0-Tesla MR system (Discovery MR750, General Electric, Milwaukee, WI, USA). A tight and comfortable foam pad was utilized to reduce head motion, and earplugs were used to minimize scanner noise. Sagittal 3D T1-weighted images were obtained by utilizing a brain volume sequence. Main parameters include: repetition time/echo time = 8.16/3.18 ms; inversion time = 450 ms; flip angle = 12 degree; field of view = 256 mm × 256 mm; matrix = 256 × 256; slice thickness = 1 mm; no gap; 188 sagittal slices.

### Brain volume calculation

**FIGURE 1:**
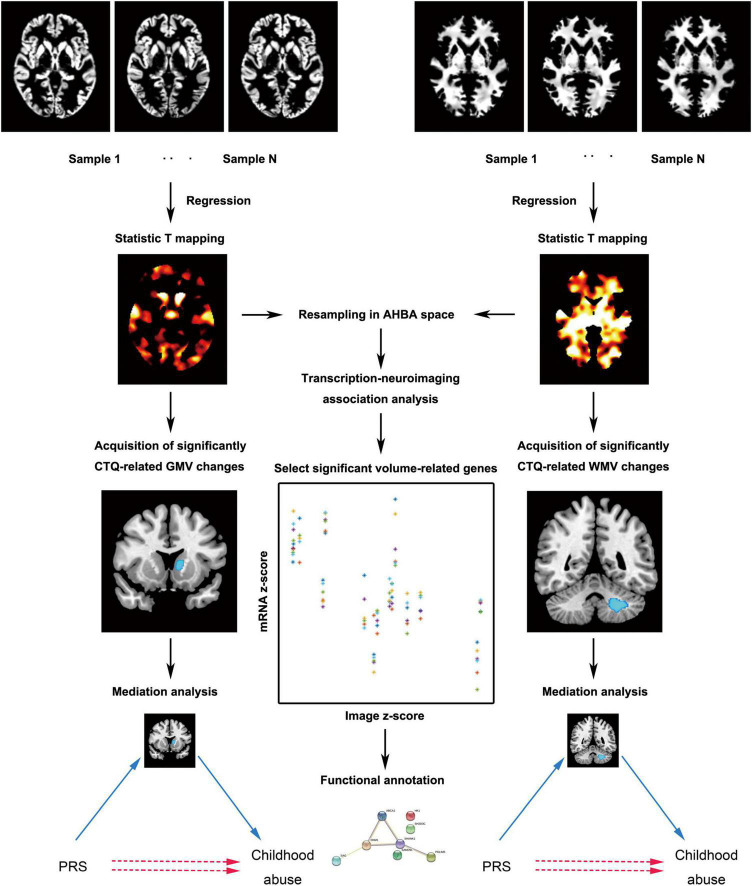
The main analytic framework of this study. AHBA, Allen Human Brain Database; CTQ, Childhood Trauma Questionnaire; GMV, gray matter volume; PRS, polygenic risk score; WMV, white matter volume.

We used the standard pipeline of the CAT12 toolbox^2^ (RRID:SCR_019184) which runs within SPM12^3^ to calculate gray matter volume (GMV) and white matter volume (WMV). First of all, the tissue probability maps of SPM12 were used for the initial spatial registration and segmentation. After correcting for bias-field inhomogeneity and affine registration to standard space, T1-weighted images were segmented into cerebrospinal fluid, gray matter, and white matter. By means of the DARTEL technique, the segmented images were further normalized using the diffeomorphic anatomical registration (Ashburner, 2007). Then normalized images were resampled to a voxel size of 1.5 mm × 1.5 mm × 1.5 mm. A partial volume effect label image volume for quality control was also written. The Jacobian determinant for each voxel was written in normalized space. We also estimated total intracranial volume as a covariate for all analyses to correct different brain sizes. Finally, the modulated normalized gray matter and white matter segments were smoothed with a Gaussian kernel of 8 mm × 8 mm × 8 mm full-width at half maximum. Next, the analysis flowchart of this study is presented in Figure 1.

### Statistical analysis

We explored the correlations between the abuse score and the whole-brain GMV/WMV using a multiple linear regression model in SPM12. Sex, education level, age, and total intracranial volume were controlled as covariates. For each cluster on the basis of the whole-brain findings (voxel-wise *P* < 0.001 uncorrected; cluster size > 10 voxels), the resultant statistical parametric maps were thresholded at cluster-level corrected *P*_FWE_ < 0.05. If the results cannot pass the strict whole-brain family-wise error (FWE) correction, a 30-mm spherical dimension centered at the peak location of the cluster was placed around each cluster, and the small-volume FWE correction (*P* < 0.05) was used as an alternative reference to correct for multiple comparisons. Additionally, statistical analyses for the demographic data were performed using Statistical Package for the Social Sciences version 18.0. The normal distribution of behavioral and imaging data was tested by Kolmogorov–Smirnov test. The reliability of the scales was checked by Cronbach’s alpha coefficient. Two-sample *T*-test was applied to test gender differences in PRS, abuse scores, age, and education level.

### Mediation analysis

In this study, a three-variable mediation model of the SPSS macro^4^ was used to determine the relationship among PRS score, brain volume alterations, and abuse score, which may improve our understanding of the relationship between inherited risk and acquired behavior, even when they appear to lack a direct correlation. We defined the PRS score as the independent variable X, the GMV/WMV alterations as the mediator variable M, and the abuse score as the dependent variable Y. The bootstrapping method was adopted to assess the significance of the mediating effect. According to the distribution of the indirect effect after 5000 bias-corrected bootstrapping, we calculated 95% confidence intervals for the effect. If zero was not within the 95% confidence interval of the result, we concluded that there was a significant mediating effect.

### Imaging-genomic correlation analysis

When evaluating the relationship between GMV/WMV and abuse score respectively, a whole-brain statistic T mapping was generated in the multiple linear regression. After normalizing the whole-brain statistic T mapping to z-scores and resampling in the genomic space independently for each donor, we explored the correlation patterns between the T mapping and mRNA gene expression profiles derived from the AHBA in MENGA software platform (Rizzo et al., 2016). A process similar to the following analysis was described in detail in previous studies (Rizzo et al., 2016; Tian et al., 2021). The main menu options included: (a) we used the default region list, which corresponds to the simplified AHBA coarse resolution level in MENGA; (b) the default mask obtained from the FSL MNI ICBM152 template was selected and confined to the left hemisphere to benefit from the contribution of all donors; (c) the list of genes included 883 risk genes detected by our whole exome sequencing within the threshold (*P*_T_ = 0.0354).

The univariate cross-correlation analysis included weighted regression of each donor’s mRNA and image data. The resulting squared Pearson’s correlation coefficients (*R*^2^) were displayed in a matrix of 6 × 1 elements and reported as the mean and standard deviation across the different donors in the summary statistics. The correlation was significant for a correlation coefficient *R*^2^ > 0.25 after Bonferroni correction (*P* < 0.05).

The multivariate cross-correlation included weighted multiple regression between the mRNA data of the different donors and the average values of image areas across donors. The fitting performance of the model was defined by the adjusted coefficient of determination (*R*^2^_adj_) that represents the amount of the total image variability explained by the genomic data. The multivariate analysis was completed with the calculation of the chance-likelihood, which represents a measure of cross-correlation reliability. If the chance-likelihood value is less than 5%, the correlation is highly reliable.

### Functional annotation

For genes consistently associated with GMV/WMV alterations, we further performed enrichment analysis by Toppogene^5^ to explore their highly enriched biological pathways based on gene ontology (GO) database. The Benjamini and Hochberg method for false discovery rate (FDR-BH correction) (*P* < 0.05) was used for multiple comparisons correction. We also created protein-protein interaction (PPI) networks by STRING^6^ to understand the functional relationship between different proteins.

## Results

### Demographic and behavioral characteristics

Among the 216 participants, 177 completed the whole exome sequencing, and their demographic information and behavioral characteristics are summarized in Table 1. The Cronbach’s alpha value for the whole scale was satisfactory (α = 0.709). Imaging characteristics and behavioral scores that are set as the dependent variable satisfied normal distribution. There were no significant gender differences in PRS, abuse scores, age, and education level.

**TABLE 1:** Demographic and behavioral characteristics of the sample.

Demographics	Mean (SD)	Range
Gender (female/male)	130/47	NA
Age (years)	23.97 (1.88)	20–30
Education level (years)	17.65 (1.60)	13–22
Emotional abuse	6.12 (1.42)	5–11
Sexual abuse	5.23 (0.64)	5–9
Physical abuse	5.50 (1.24)	5–14
Overall childhood abuse	16.85 (2.47)	15–30
Best-fit PRS	0.37 (0.27)	−0.31 to 1.09
Total PRS	1.39 (0.58)	0.03–3.64

NA, not applicable; PRS, polygenic risk score; SD, standard deviation.

### Gray matter volume alterations related to abuse score

**FIGURE 2:**
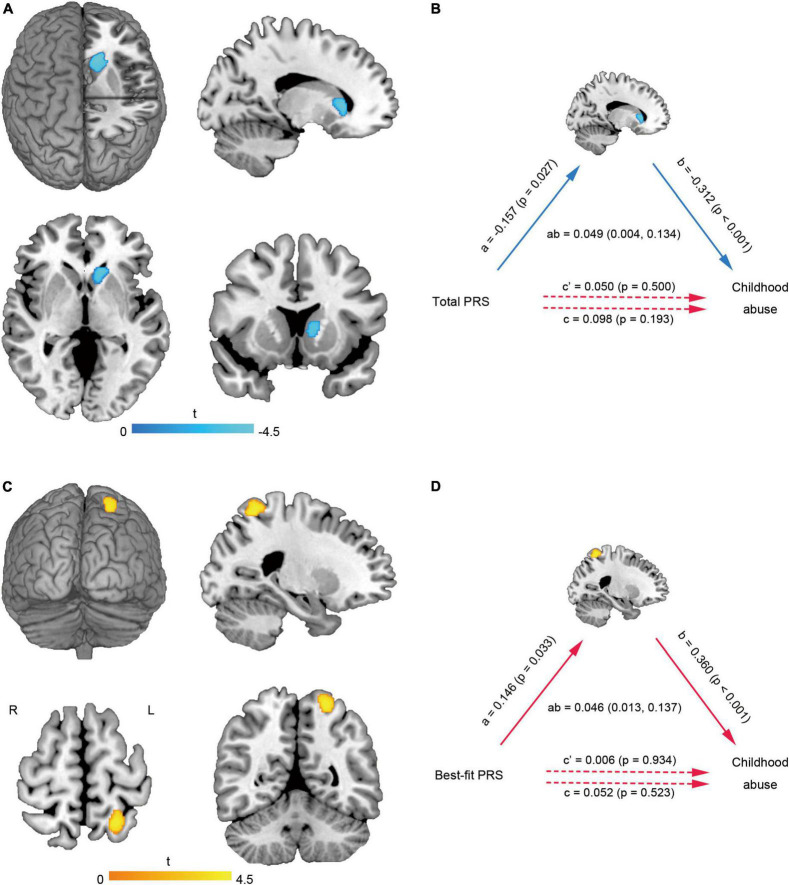
Brain gray matter alterations related to childhood abuse and mediation analysis. The GMV of left caudate **(A)** was negatively correlated with the total abuse score. The left caudate changes mediated the effect of total PRS on childhood abuse score **(B)**. The GMV of left superior parietal lobule **(C)** was positively correlated with the total abuse score. The left superior parietal lobule changes mediated the effect of best-fit PRS on childhood abuse score **(D)**. GMV, gray matter volume; L, left; PRS, polygenic risk score; R, right.

Multiple linear regression showed that the GMV of left caudate (MNI coordinate: −12 24 5, 540 voxels, *T* = −4.47) (Figure 2A) was negatively correlated with the total abuse score (*P*_uncorr_ = 0.017, *P*_FWE–corr_ = 0.113, small-volume *P*_FWE–corr_ = 0.009). Mediation analysis further found that there was no significant direct correlation between the total PRS and childhood abuse, but there was significant indirect correlation mediated by the left caudate change (Figure 2B). The GMV of left superior parietal lobule (MNI coordinate: −23 −56 63, 372 voxels, *T* = 4.44) (Figure 2C) was positively correlated with the total abuse score (*P*_uncorr_ = 0.041, *P*_FWE–corr_ = 0.256, small-volume *P*_FWE–corr_ = 0.022). Mediation analysis further found that there was no significant direct correlation between the best-fit PRS and childhood abuse, but there was significant indirect correlation mediated by the left superior parietal lobule change (Figure 2D).

### White matter volume alterations related to abuse score

**FIGURE 3:**
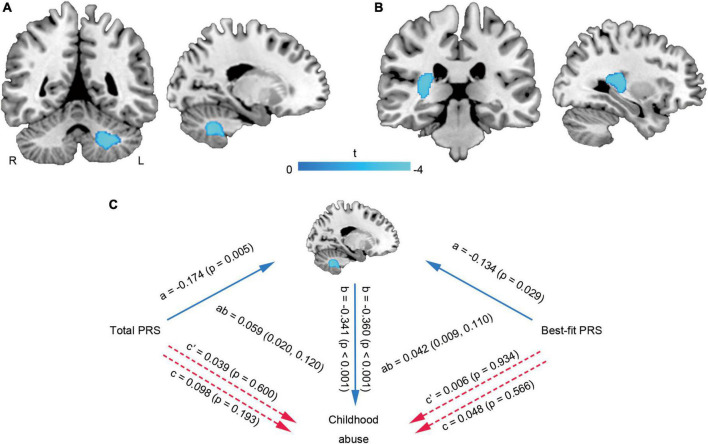
Brain white matter alterations related to childhood abuse and mediation analysis. The WMVs of left cerebellum cluster **(A)** and right temporal lobe-basal ganglia region **(B)** were negatively correlated with the total abuse score. The cerebellar WMV-change mediated the effects of PRS on childhood abuse score **(C)**. L, left; PRS, polygenic risk score; R, right; WMV, white matter volume.

Multiple linear regression showed that the WMVs of left cerebellum cluster (MNI coordinate: −21 −56 −39, 923 voxels, *T* = −3.90, P_FWE–corr_ = 0.019) (Figure 3A) and right temporal lobe-basal ganglia region (MNI coordinate: 26 −33 12, 895 voxels, *T* = −4.08, *P*_FWE–corr_ = 0.021) (Figure 3B) were negatively correlated with the total abuse score. Mediation analysis further found that no matter whether it was the total PRS or the best-fit PRS, there were no significant direct correlations between them and childhood abuse, but there were significant indirect correlations mediated by the cerebellar WMV-change (Figure 3C).

### Imaging-genomic correlation analysis

A sample-wise spatial correlation was performed between the expression level of each risk gene and childhood abuse-related GMV/WMV changes. We found that there were 62 genes (Supplementary Table 1) significantly associated with childhood abuse-related WMV changes (univariate Pearson’s correlation coefficient *R*^2^ > 0.25, chance-likelihood of the multivariate correlation coefficient < 5%), while only the expression level of RADIL was significantly associated with childhood abuse-related GMV changes (*R*^2^ = 0.257, chance-likelihood = 3%).

### Enrichment analysis

**FIGURE 4:**
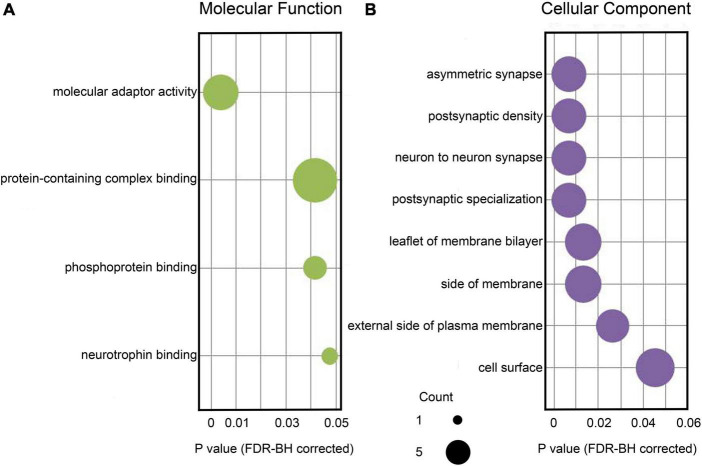
Enrichment analysis based on the identified 62 genes associated with brain WMV alterations. **(A)** Significant GO items of molecular function. **(B)** Significant GO items of cellular component. The x-axis displays corrected *P*-values for each GO item. The size of each sphere shows the number of genes overlapping with each GO item. GO, gene ontology; WMV, white matter volume.

Table 2 summarizes biological functions of the RADIL gene associated with GMV alterations. The 62 identified genes associated with WMV alterations had shown significant enrichment for molecular functions and cellular components (FDR-BH *P* < 0.05 corrected) (Figures 4A,B).

**TABLE 2:** Functional relevance of the RADIL gene based on gene ontology (GO) database.

Category	ID	Name
Molecular function	GO:0051020	GTPase binding
Biological process	GO:0001755	Neural crest cell migration
	GO:0014032	Neural crest cell development
	GO:0014031	Mesenchymal cell development
	GO:0048864	Stem cell development
	GO:0014033	Neural crest cell differentiation
	GO:0034446	Substrate adhesion-dependent cell spreading
	GO:0048762	Mesenchymal cell differentiation
	GO:0048863	Stem cell differentiation
	GO:0060485	Mesenchyme development
	GO:0031589	Cell-substrate adhesion
	GO:0001667	Ameboidal-type cell migration
	GO:0000904	Cell morphogenesis involved in differentiation
Cellular component	GO:0005874	Microtubule

### Protein-protein interaction network analysis

**FIGURE 5:**
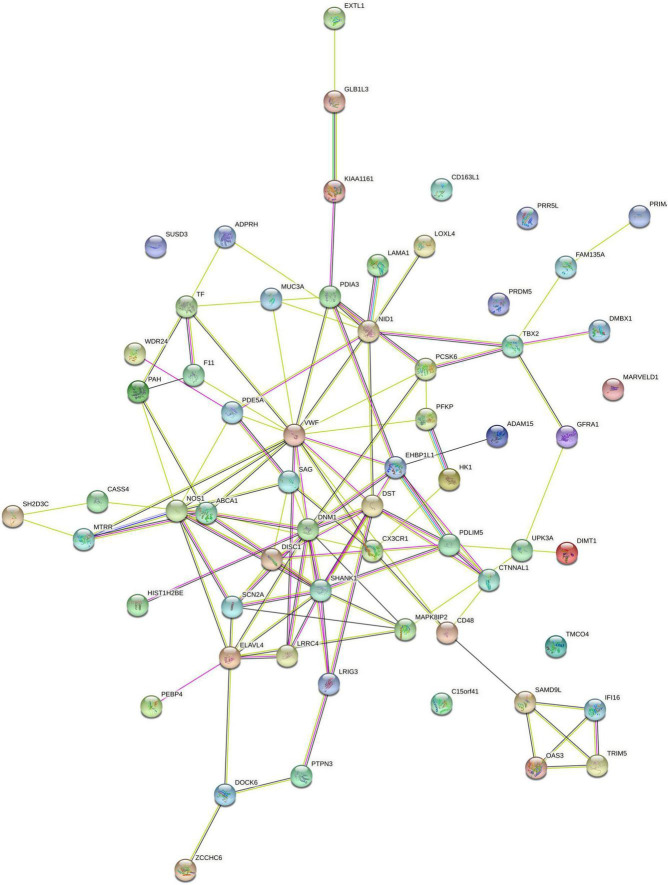
PPI networks constructed by 62 identified genes related to brain WMV changes. PPI, protein-protein interaction; WMV, white matter volume.

Based on 62 genes significantly correlated with WMV alterations, we conducted PPI network analysis and identified a network consisting of 55 connected proteins and 114 edges (PPI enrichment *p*-value: 0.060) (Figure 5). According to the degree of nodes (Supplementary Figure 1), the proteins coded by VWF, SHANK1, DNM1, and NOS1 were hub proteins in the PPI network. According to their co-expression values (Supplementary Figure 2), SAMD9L and OAS3 genes, SAMD9L and IFI16 genes, PAH and F11 genes were observed to be highly correlated in protein expression, respectively.

## Discussion

As far as we know, this is the first study that combined PRS with neuroimaging to assess the development of self-reported childhood abuse, and further identified risk genes whose expressions were related to brain volume changes. First of all, we found that there was a significant correlation between childhood abuse and brain volume changes. Moreover, the volume changes of brain regions mediated the influence of polygenic risk on self-reported childhood abuse. Further, we analyzed spatial correlations between the expressions of risk genes and the brain volume changes. We found 63 risk genes whose expression levels were significantly associated with childhood abuse-related brain volume changes. These genes are involved in biological processes, such as neural development, synaptic transmission, and cell construction. Combined with the plasticity of brain morphology, our work helps the public to understand the neuromechanism of childhood abuse from the different dimensions of molecular level.

From the perspective of neuroimaging, our findings indicate an evident role of brain volume alterations in the occurrence of childhood abuse. The caudate nucleus functions in reward-processing, motivation, memory, learning, and emotional interaction (Driscoll et al., 2022). Previous studies have demonstrated that abused subjects are sensitive to rewards in regulating cognitive control (Kiyar et al., 2021). An imaging study also showed that the change of GMV in the caudate nucleus was related to childhood abuse (Wang J. et al., 2021). The superior parietal lobule participates in the integration of somatosensory and visual space, and plays additional roles in written language, attention, and working memory (Lin et al., 2021). An exploratory cortical analysis showed that childhood maltreatment was associated with altered morphology development of superior parietal region during adolescence (Whittle et al., 2013). It is agreed that the cerebellum is important in emotional and cognitive development. Brain imaging research has provided evidence that differences in cerebellar structure are associated with childhood abuse (McCrory et al., 2012). Moreover, cerebellar volumes were positively correlated with age of onset of the childhood maltreatment and negatively correlated with the duration of the maltreatment that led to psychopathology (De Bellis and Kuchibhatla, 2006). Although relatively few studies have focused on white matter abnormalities related to childhood abuse, it is known that widespread changes in white matter microstructure connecting the frontal, temporal, and occipital cortices mediate emotional and cognitive control in childhood abuse (Lim et al., 2019). Taken together, consistent with previous findings to a certain extent, we found that volume abnormalities in basal ganglia, cerebellum, parietal lobe, and temporal regions exhibited strong associations with childhood abuse, which may be the pathological basis of self-reported childhood abuse.

Identifying the important role of genetic risk can provide more information about the neural mechanisms underlying gene-brain-behavior associations. Following this idea, several previous studies have linked non-invasive neuroimaging techniques with PRS to determine the brain changes related to the genetic risk of mental and psychological behaviors (Hermosillo et al., 2020; Wang J. et al., 2021). These genetic imaging studies have laid a solid foundation for exploring the pathogenesis of mental illness. In this study, although there was no significant direct correlation between PRS and childhood abuse, we found that PRS affects self-reported childhood abuse by modulating brain volumes. Mediation effects explain the complexity of the relationship between genes, brain, and behaviors, and to some extent reveal the internal pathogenesis of childhood abuse. The altered brain development may be one mechanism by which polygenetic variation has continuing effects on childhood maltreatment. Multi-gene participation is more in line with the intricate genetic mechanisms of brain development and the complicated psychopathological traits of childhood abuse. Our findings also highlight the significance of early interventions for individuals who have a high genetic risk for childhood abuse.

The complex effects of genes on brain and behavior are also assumed to be controlled by gene expression. In this manuscript, we confirmed this hypothesis by showing the significant spatial correlations between childhood abuse-GMV/WMV correlation maps and the expression data of risk genes. As there is no large sample of childhood maltreatment who have both neuroimaging and gene data of the brain, we can only indirectly identify the risk genes whose expression levels are related to brain changes of abused children through the AHBA database. Although our results have some inaccuracies, we still believe that this method can provide some interesting and meaningful information. On one hand, the expression level of RADIL was significantly associated with childhood abuse-related GMV changes. RADIL is an important player for cell adhesion, cell migration, and the epithelial-mesenchymal transition (Smolen et al., 2007; Ahmed et al., 2010; Choi et al., 2021). It is necessary for the development of nerve cells. On the other hand, our results indicated that WMV alterations were related to the joint-effects of many childhood abuse-related genes. Enrichment and PPI network analyses linked these genes with nerve development, synaptic transmission, and cell construction. Taking several genes encoding hub proteins as examples, SHANK1 plays a role in the structural and functional organization of the dendritic spine and synaptic junction. Recent human genetic studies reported that SHANK family genes were pathogenic genes for idiopathic autism spectrum disorders (Jiang and Ehlers, 2013). NOS1 produces nitric oxide, which is a messenger molecule in the brain and peripheral nervous system. The NOS1 gene is functionally associated with cognitive and neuropsychiatric symptoms (Ahmed et al., 2017). DNM1 is a dynamin-related gene. Pathogenic DNM1 gene variant presents with developmental delay and neurologic disorders (Deng et al., 2016; Li et al., 2019). VWF is important in the maintenance of hemostasis. The effects of abnormal VWF expression on cerebral blood circulation may induce neuronal death, and is associated with poor long-term cognitive outcomes even in the young (Pedersen et al., 2018). To sum up, our work preliminarily describes the impact these risk genes expression may have on the pathophysiology of the brain related to childhood abuse.

There are several limitations of this research that have to be mentioned. First of all, it is necessary to mention the restriction on blood background. We computed PRS for childhood abuse based on GWAS analyses in European ancestry, as there are no such GWAS analyses in Asian ancestry. Although it is an indirect method to estimate polygenic risk in the Chinese sample, many articles have used this indirect method to explore the risk of mental illness in Asian samples and reported many important findings (Wang et al., 2018, 2020; Wang J. et al., 2021). However, racial differences can’t be ignored, and we look forward to establishing an Asian database to verify these findings in the future. Second, we cannot make causal statements about the relationship of volume changes and expression levels, because the two data sources come from different samples. The gene expression of AHBA donors cannot fully and accurately reflect the actual gene expression of investigated subjects. But this research method can still prompt some preliminary and meaningful findings (Richiardi et al., 2015; Ji et al., 2021). Another possible limitation is the subjective response bias in the self-report questionnaire. We lack multiple interviews or reviews to verify the data, in order to protect the privacy of participants. Finally, for PRS and imaging-genomic correlation analyses, our sample size is still too small and a large database is needed for further exploration and verification in the future.

Combined with neuroimaging, the present findings yield insights into the crucial roles of polygenic risk and gene expression in the occurrence of childhood abuse. The cumulative effect of high-risk gene variations and abnormal expression of genes related to nervous system development may be molecular mechanisms accounting for brain volume changes, which is closely related to the development of childhood abuse. Childhood trauma exposure is common and far-reaching, but it can usually be prevented after understanding its genetic mechanisms. Our findings may not only help to understand the occurrence of childhood abuse, but also eventually provide a clear goal for public health efforts focusing on children to formulate timely and effective early prevention strategies for ameliorating the incidence rate.

## Data availability statement

The datasets used and/or analyzed during the current study are available from the corresponding author on reasonable request. Requests to access these datasets should be directed to WZ, zhuwenzhen8612@163.com.

## Ethics statement

The studies involving human participants were reviewed and approved by Tongji Hospital, Tongji Medical College, Huazhong University of Science and Technology. The patients/participants provided their written informed consent to participate in this study.

## Author contributions

TT, YL, and DL made substantial contributions to conception and design, acquisition of the data, and analysis and interpretation of the data. GZ, JW, JL, CW, JF, DW, YZ, HZ, and YQ made substantial contributions to acquisition, analysis, and interpretation of the data. TT, DL, and WZ were involved in drafting the manuscript and revising it critically for important intellectual content. All authors agreed to be accountable for the content of the work and approved the submitted version.

## Footnotes

1
http://human.brain-map.org

2
http://dbm.neuro.uni-jena.de/cat

3
http://www.fil.ion.ucl.ac.uk/spm

4
http://www.processmacro.org/index.html

5
https://toppgene.cchmc.org/

6
https://string-db.org/
