# Supplementary material for: Genetic influence on brain volume alterations related to self-reported childhood abuse
Source: Front Neurosci. 2022 Sep 20;16:1019718. doi: 10.3389/fnins.2022.1019718 (PMC9530554; doi:10.3389/fnins.2022.1019718)
Supplement: Supplementary file 1 [file Presentation_1.pdf]

Supplementary Material

1 Supplementary Data

Recruitment criteria included: (a) all participants had no history of severe head trauma, genetic psychiatric or neurological illness, psychiatric treatment, poisoning or metabolic diseases, or drug or alcohol abuse; (b) no contraindications for MRI examination; (c) Chinese Han populations; and (d) strongly right-handed.

Genomic DNA was extracted from each subject using DNeasy Blood & Tissue Kit (QIAGEN). Exome capture was performed using the SureSelect Human All Exon V6 (Agilent Technologies) according to the manufacturer's instructions. The quantity of libraries was assessed by Qubit® 2.0 Fluoromete. The quality and size of libraries were measured by 2100 Bioanalyzer High Sensitivity DNA Assay according to the reagent kit guide. For Illumina sequencing, the qualified libraries were applied to 2×150 bp paired-end sequencing on Illumina HiSeq X-ten platform (Illumina). FASTQ files were aligned to human reference genome by BWA v0.7.13. The aligned files (BAM format files) were sorted by samtools (1.3) firstly, then duplicates were flagged by using Picard (2.2.4). By using GATK v3.5, reads were locally realigned and base qualities were recalibrated. Finally, mapping statistics, including coverage and depth, were generated from recalibrated files by BEDTools (2.16.1) and in-house perl/python scripts. Variants (single-nucleotide variations and indels) were genotyped from recalibrated BAM files using the multi-sample processing mode of the Unified Genotyper tool from the GATK. Then VQSR (Variant Quality Score Recalibration) was used to reduce false positive of variant calling. Copy Number Variation was identified by XHMM (eXome Hidden Markov Model) v1.0. Single-nucleotide variations and indels were annotated using ANNOVAR software against multiple databases including HGVS variant description, population frequency, disease or phenotype and variant functional prediction. SNPs' data quality control procedures were illustrated using the whole-genome association analysis toolset PLINK v1.07.

2 Supplementary Tables

Supplementary Table 1. Correlations between expression values of the 62 discovered genes and childhood abuse-related WMV changes.

Gene symbol	Gene ID	Univariate cross-correlation	Multivariate cross-correlation
		R^2	Chance-likelihood
ABCA1 (ATP binding cassette subfamily A member 1)	19	0.424	4%
ADAM15 (ADAM metallopeptidase domain 15)	8751	0.403	4%
ADPRH (ADP-ribosylarginine hydrolase)	141	0.411	1%
CD163L1 (CD163 molecule like 1)	283316	0.358	2%
CTNNAL1 (catenin alpha like 1)	8727	0.267	4%
DNM1 (dynamin 1)	1759	0.517	0%
C15orf41 (chromosome 15 open reading frame 41)	84529	0.302	3%
CASS4 (Cas scaffold protein family member 4)	57091	0.341	0%
CD48 (CD48 molecule)	962	0.325	1%
CX3CR1 (C-X3-C motif chemokine receptor 1)	1524	0.338	0%
DIMT1 (DIM1 rRNA methyltransferase and ribosome maturation factor)	27292	0.334	0%
DISC1 (DISC1 scaffold protein)	27185	0.376	4%
DMBX1 (diencephalon/mesencephalon homeobox 1)	127343	0.279	2%
DOCK6 (dedicator of cytokinesis 6)	57572	0.389	1%
DST (dystonin)	667	0.372	3%
F11 (coagulation factor XI)	2160	0.342	1%
GFRA1 (GDNF family receptor alpha 1)	2674	0.394	3%
HIST1H2BE (Histone cluster 1 h2b family member e)	8344	0.276	1%
EHBP1L1 (EH domain binding protein 1 like 1)	254102	0.268	2%
ELAVL4 (ELAV like RNA binding protein 4)	1996	0.261	2%

EXTL1 (exostosin like glycosyltransferase 1)	2134	0.260	4%
FAM135A (family with sequence similarity 135 member A)	57579	0.365	4%
GLB1L3 (galactosidase beta 1 like 3)	112937	0.367	2%
HK1 (hexokinase 1)	3098	0.463	1%
LRRC4 (leucine rich repeat containing 4)	64101	0.394	0%
MAPK8IP2 (mitogen-activated protein kinase 8 interacting protein 2)	23542	0.330	1%
PAH (phenylalanine hydroxylase)	5053	0.318	3%
PFKP (phosphofructokinase, platelet)	5214	0.320	2%
PTPN3 (protein tyrosine phosphatase non-receptor type 3)	5774	0.253	4%
IFI16 (interferon gamma inducible protein 16)	3428	0.305	4%
KIAA1161 (myogenesis regulating glycosidase)	57462	0.379	1%
LAMA1 (laminin subunit alpha 1)	284217	0.291	3%
LOXL4 (lysyl oxidase like 4)	84171	0.343	2%
LRIG3 (leucine rich repeats and immunoglobulin like domains 3)	121227	0.272	3%
MARVELD1 (MARVEL domain containing 1)	83742	0.416	0%
MTRR (5-methyltetrahydrofolate-homocysteine methyltransferase reductase)	4552	0.313	1%
MUC3A (mucin 3A, cell surface associated)	4584	0.318	0%
NID1 (nidogen 1)	4811	0.358	0%
NOS1 (nitric oxide synthase 1)	4842	0.291	3%
OAS3 (2'-5'-oligoadenylate synthetase 3)	4940	0.326	3%
PCSK6 (proprotein convertase subtilisin/kexin type 6)	5046	0.297	3%
PDE5A (phosphodiesterase 5A)	8654	0.371	3%
PDIA3 (protein disulfide isomerase family A member 3)	2923	0.381	0%
PDLIM5 (PDZ and LIM domain 5)	10611	0.442	2%
PEBP4 (phosphatidylethanolamine binding protein 4)	157310	0.360	1%
PRDM5 (PR/SET domain 5)	11107	0.336	2%

PRIMA1 (proline rich membrane anchor 1)	145270	0.315	1%
PRR5L (proline rich 5 like)	79899	0.261	1%
SAG (S-antigen visual arrestin)	6295	0.442	1%
SAMD9L (sterile alpha motif domain containing 9 like)	219285	0.428	0%
SUSD3 (sushi domain containing 3)	203328	0.332	2%
TBX2 (T-box transcription factor 2)	6909	0.375	1%
TF (transferrin)	7018	0.261	1%
TMCO4 (transmembrane and coiled-coil domains 4)	255104	0.284	3%
TRIM5 (tripartite motif containing 5)	85363	0.348	3%
VWF (von Willebrand factor)	7450	0.336	0%
ZCCHC6 (Zinc finger cchc-type containing 6)	79670	0.357	1%
SCN2A (sodium voltage-gated channel alpha subunit 2)	6326	0.328	4%
SH2D3C (SH2 domain containing 3C)	10044	0.432	1%
SHANK1 (SH3 and multiple ankyrin repeat domains 1)	50944	0.499	0%
UPK3A (uroplakin 3A)	7380	0.366	0%
WDR24 (WD repeat domain 24)	84219	0.270	0%

3 Supplementary Figures

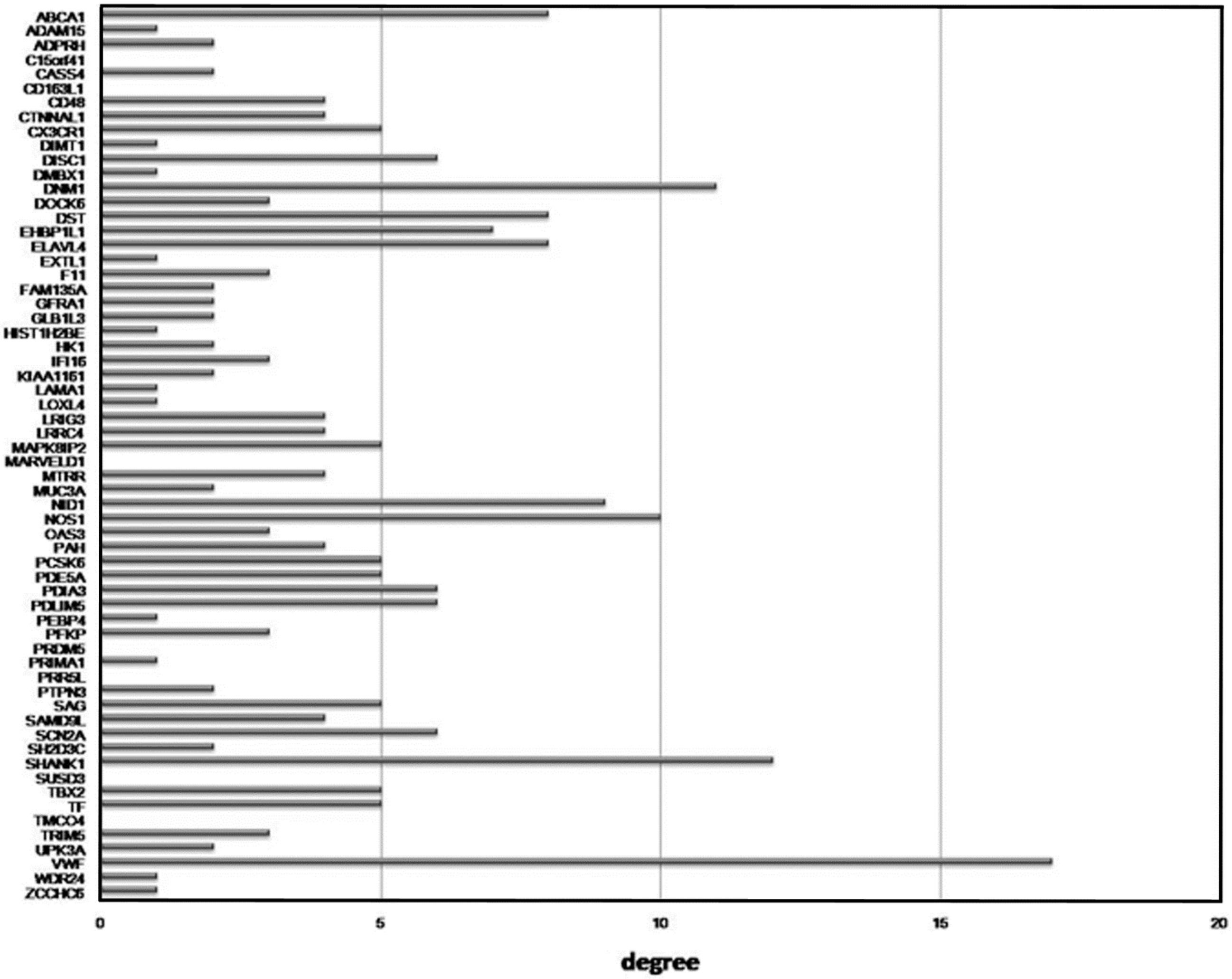

Supplementary Figure 1. Degree of nodes in protein-protein interaction (PPI) network.

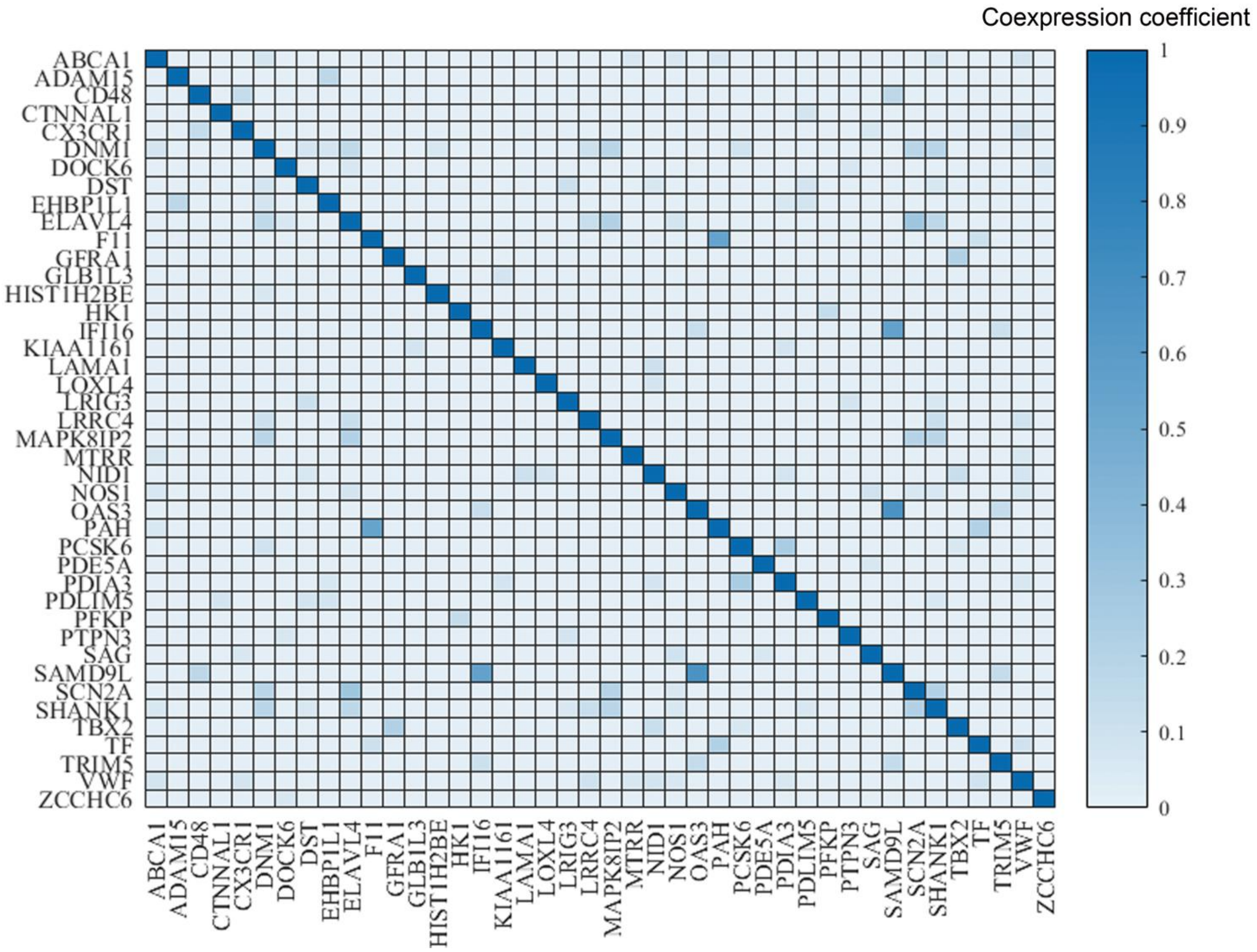

Supplementary Figure 2. Co-expression values of genes in protein-protein interaction (PPI) network.
